# Recommendation of antibiotic prophylaxis in orthognathic surgery according to current microbial resistance, a retrospective analysis of 100 operated cases

**DOI:** 10.4317/medoral.27519

**Published:** 2025-08-16

**Authors:** Pedro Sole, Pedro Tapia, Sebastián Mordoh, Melissa Carvajal, Gustavo Matus-Miranda, Benjamin Ossandón-Zuñiga

**Affiliations:** 1 ORCID: 0009-0007-2277-0275. Department of Maxillofacial Surgery, ICEO Estoril Clinic, Santiago, Chile; 2 ORCID: 0000-0002-3849-8700. Department of Maxillofacial Surgery, Franco Ravera Z Hospital, Rancagua, Chile; 3 ORCID: 0000-0002-3849-8700. Department of Maxillofacial Surgery, University of Desarrollo, Santiago, Chile; 4 ORCID: 0000-0002-4458-1981. Department of Maxillofacial Surgery, University of Desarrollo, Santiago, Chile; 5 ORCID: 0000-0001-5256-5132. Department of Maxillofacial Surgery, University of Desarrollo, Santiago, Chile; 6 ORCID: 0000-0003-0966-7576. Resident in Oral and Maxillofacial Surgery, University of Chile, Chile; 7 ORCID: 0000-0001-5039-9337. Doctor of Dental Surgery, Universidad de Chile, Chile

## Abstract

**Background:**

Maxillofacial infections (MIs), particularly surgical site infections (SSIs) following orthognathic surgery, represent a significant clinical concern due to their potential severity and impact on treatment outcomes. Although antibiotic prophylaxis is standard practice, growing bacterial resistance-especially in penicillin-allergic patients-challenges the effectiveness of alternative regimens such as clindamycin.

**Material and Methods:**

This retrospective study analyzed 100 consecutive patients who underwent orthognathic surgery between 2022 and 2023, performed by the same surgical team. All patients received standardized perioperative care, including hospitalization and intravenous (IV) antibiotic prophylaxis with cefazolin or clindamycin for those allergic to penicillin. The incidence of SSIs was evaluated and correlated with the type of antibiotic used. Microbial cultures and antibiograms were obtained from infected cases requiring surgical wound revision.

**Results:**

Of the 100 patients, 98 received prophylactic cefazolin and showed no SSIs. The remaining 2 patients, both allergic to penicillin and treated with IV clindamycin, developed SSIs within the first postoperative week. Both cases required surgical drainage, hospitalization, and culture-based antibiotic therapy. Pathogens isolated included Streptococcus mitis, S. oralis, S. constellatus, and Haemophilus parainfluenzae, all resistant to clindamycin and erythromycin but sensitive to beta-lactams and fluoroquinolones. Both patients responded favorably to ciprofloxacin and metronidazole.

**Conclusions:**

This study highlights a significant risk of infection associated with clindamycin prophylaxis in penicillin-allergic patients undergoing orthognathic surgery. Cefazolin proved effective in preventing SSIs. These findings underscore the urgent need for updated, evidence-based prophylactic protocols in maxillofacial surgery, particularly for patients with beta-lactam allergies.

## Introduction

Maxillofacial infections (MIs) are considered a public health problem due to their great potential for dissemination to important and vital anatomical structures. MIs are characterized as polymicrobial, endogenous, opportunistic, dynamic, and mixed (facultative anaerobic streptococcal and strict anaerobic bacteria) (1 - 3). MIs have a varied etiology, with both odontogenic and non-odontogenic causes. Of MIs, surgical site infections (SSIs) in orthognathic surgery stand out. Data on the infection rates in orthognathic surgery vary greatly, from 1.4% to 33.4%. They jeopardize the surgical outcome and represent a significant problem for the patient and the treating physician (4 , 5).

Generally, MIs respond well to surgical treatment based on incisions, drains, and antibiotherapy. However, the progression and dissemination of infections depends on factors such as immunosuppression states, the anatomical location of the infection, the virulence of the pathogens, microbial resistance, and adequate medicosurgical management. When dissemination occurs, deep cervical spaces may be involved and risk compromising the airway or causing mediastinitis and thrombosis of the cavernous sinus. Systemic spread of the infection may lead to organ dysfunction, inflammatory systemic response syndrome, or sepsis, and it could be fatal (5 - 7).

Since the clinical introduction of penicillin in 1940, infectious pathogens have evolved, and antibiotic therapy has become increasingly complex (8). Therefore, despite our knowledge of the maxillofacial microflora, there are great difficulties in treating these infections due to the emergence of resistant strains of bacteria, including viridans streptococci, Staphylococcus spp., and Prevotella spp., among others. This increase in bacterial resistance has led to an increase in morbidity and mortality among patients with head and neck infections (9 , 10).

The aim of this retrospective study was to determine the MI rate in a series of 100 patients who received orthognathic surgery by the same surgical team over a 1-year period based on the type of antibiotic used for prophylaxis. This study also discusses the choice of antibiotic prophylaxis in orthognathic surgery and how it has been affected by bacterial resistance.

## Material and Methods

A retrospective analysis of 100 patients undergoing orthognathic surgery was performed. Patient information was collected from the surgical team's electronic database, which included patients with dentofacial anomalies and malocclusions who underwent orthognathic surgery in private practice between 2022 and 2023. Only patients with complete clinical and radiological documentation were included, as well as patients who received bilateral sagittal ramus sagittal osteotomy (BSSO), Le Fort I osteotomy, or bimaxillary surgery with or without genioplasty. All procedures were performed after adequate orthodontic preparation. The surgeries were performed by the same surgeon during the observation period. Osteosynthesis with titanium mini-plates for rigid internal fixation was performed in all cases. All patients completed 3 days of postoperative hospitalization with endovenous (EV) antibiotic therapy. The patients received regular postoperative surgical follow-up in conjunction with postoperative orthodontic treatment. The protocol consisted of a surgical follow-up at 1 week, 1 month, 3 months, 6 months, and 1 year.

The medical records, anesthesia protocols, and operative records were analyzed retrospectively. The clinical characteristics of the patients, including antibiotic allergies, preexisting conditions, age, sex, and dentofacial anomalies, were documented. The details on the surgical management, including the treatment approaches, were collected. The orthognathic surgery possibilities were: Le Fort I with or without genioplasty; BSSO with or without genioplasty; BSSO/Le Fort I with or without genioplasty.

Cefazolin 1 g EV was used as infection prophylaxis; clindamycin 600 mg EV was administered to patients allergic to beta-lactams. SSIs and their management were evaluated. SSIs were defined by standardized surveillance criteria during surgical follow-up by the same surgeon. Information regarding patients with complicated postoperative infections requiring revision of the surgical wound with intraoral incision and drainage under general anesthesia as well as additional antibiotic therapy and hospitalization was recorded.

## Results

Of the 100 patients who underwent orthognathic surgery, 52% were women and 48% were men (Table 1). Of the 100 patients, 2% were allergic to penicillins (Table 2). In addition, the patients were classified according to their dentomaxillary anomaly; 59 patients were skeletal class II (59%), and 41 patients were skeletal class III (41%) (Table 3). The type of orthognathic surgery most frequently performed was bimaxillary advancement together with genioplasty.

Table 1 Table 2 Table 3

Cefazolin 1 g EV was used as intraoperative prophylaxis for 98% of the patients. In the patients allergic to penicillin (2%), clindamycin 600 mg EV was used as intraoperative prophylaxis (Table 4). Both groups of patients continued with the appropriate antibiotherapy for the time they remained hospitalized.

Table 4

**Figure 1:**
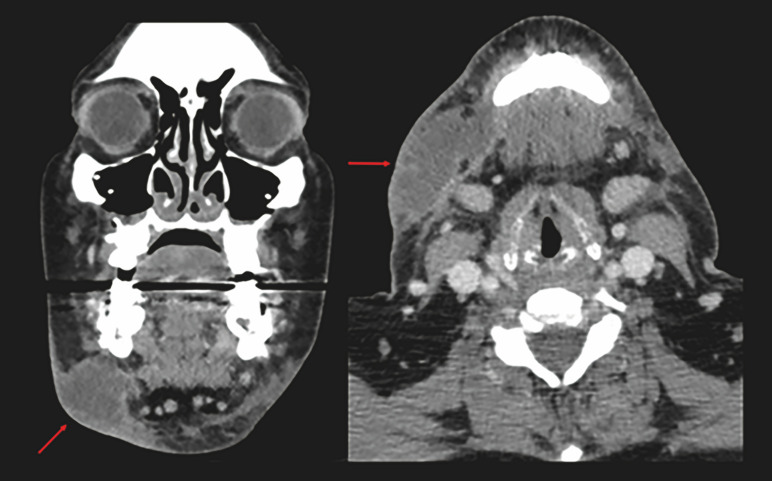
CT scan of the neck with coronal and axial contrast. Right perimandibular collectionis visualized in relation to the area with infectious process.

None of the patients who received prophylactic cefazolin developed an SSI. On the other hand, both patients who received prophylactic clindamycin developed an SSI. Both SSIs occurred during the first postoperative week and met the criteria for hospitalization and surgical revision of the wound to remove infectious material and to install a drain. Upon admission to the hospital, a neck computed tomography (CT) scan with contrast was performed (Figure 1). In addition, intraoperative culture was taken from both patients. The results showed Streptococcus mitis/Streptococcus oralis, Streptococcus constellatus, and Haemophilus parainfluenza.

1

**Figure 2:**
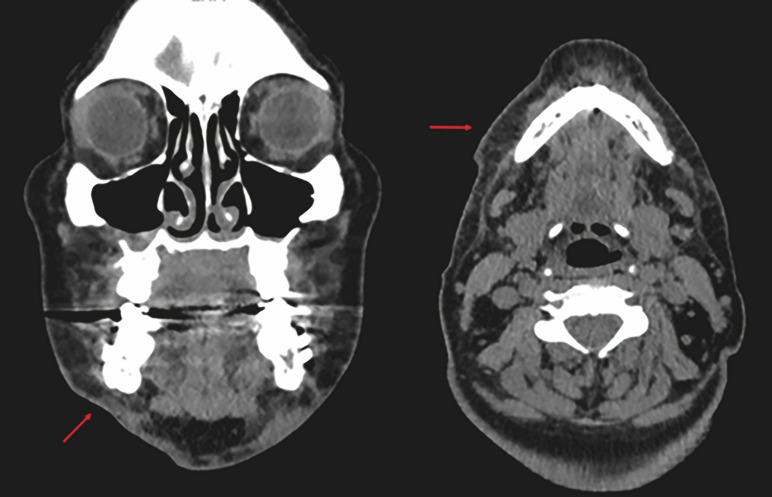
CT scan of the neck with contrast after emptying and surgical revision, coronal andaxial sections. Resolution of the infectious process without associated collections is visualized.

**Figure 3:**
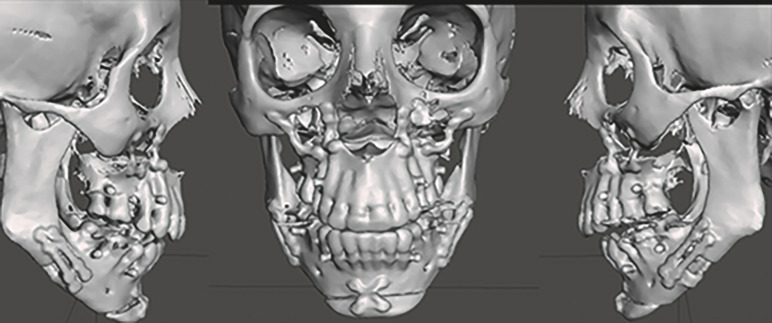
3D reconstruction of patient operated by postoperative infection, without structuralchanges, correct position of osteosynthesis.

Both antibiograms showed resistance to clindamycin and erythromycin and sensitivity to beta-lactams and fluoroquinolones. After hospitalization for 2 days and antibiotic treatment based on ciprofloxacin 500 mg every 12 h + metronidazole 500 mg every 8 h EV, the patients had a favorable outcome without relapse, and their clinical parameters were stable at hospital discharge. A neck CT scan with contrast was performed the following day and 7 days after drainage and surgical debridement (Figure 2). The SSI did not affect the postoperative surgical outcome in either patient (Figure 3).

2

3

## Discussion

A SSI in orthognathic surgery is a complication that hinders surgical progress and affects both the patient and the surgeon in its resolution. Among the variables related to SSIs are risk factors related to the patient (poor nutritional status, smoking, diabetes, and an impaired immune system, among others), the patient's anatomy, the virulence of the bacteria, and the current bacterial resistance, which is crucial in the spread of the infection. Therefore, the choice of antibiotic prophylaxis is of great importance in patients undergoing orthognathic surgery.

In addition to considering the patient's condition and comorbidities, it is important to be clear about antibiotic resistance to the different families of antibiotics. A lack of clarity has to led to an increase in SSIs due to poor antibiotic choices, especially in patients allergic to penicillin. According to this retrospective study, both patients treated with prophylactic clindamycin due to a penicillin allergy developed an SSI. In general, there is an aerobic and anaerobic polymicrobial spectrum typical of oral bacteria: viridans streptococci, Staphylococcus epidermidis, and Enterococcus faecalis. MIs can also be caused by S. mitis, S. oralis, S. constellatus, and H. parainfluenza.

According to previous studies, staphylococci are one of the four predominant bacteria isolated in oral cavity infections (11 , 12). Historically, Staphylococcus spp. have not been considered members of the oral flora nor do they play a preponderant role in the pathogenesis of head and neck infections of odontogenic origin. However, recent studies have shown that staphylococci are more frequent colonizers of the oral cavity than previously thought (11 , 12).

Heim et al. (13) reported 92 bacterial isolates in infections of odontogenic origin. Of these isolates, 79.3% were aerobes and 20.7% were anaerobes. The majority (86.3%) of the anaerobes were gram positive; the remaining 13.6% were gram negative. The study also demonstrated the presence of Candida albicans and Candida dubliniensis in about 13% of cases. The authors observed resistance to the antibiotics tested in staphylococci in 11 cases (64.7%), with low susceptibility to penicillin (58.8%). There was low susceptibility to clindamycin (83.4%), erythromycin (76.5%), and levofloxacin (83.4%). Viridans streptococci were isolated in 17 cases; they were 100% susceptible to penicillin, erythromycin, and cefazolin. The susceptibility rate to clindamycin was 84.6%. These findings show that penicillin still appears to be a very effective antibiotic in the treatment of viridans streptococci (13). Previous studies have reported that streptococci were 100% sensitive to penicillin, while only 84.6% were sensitive to clindamycin. Moreover, 78.6% of Prevotella spp. showed sensitivity to clindamycin, but all were sensitive to penicillin, gentamicin, and levofloxacin. The high susceptibility to penicillin suggests that it should be used preferentially against streptococci and Prevotella, while clindamycin should not be used as a first-line antibiotic (13 , 14).

Weise et al. (6) conducted a retrospective study of 483 patients with infections of odontogenic origin. They collected a culture from all patients and performed an antibiogram. All groups of isolated pathogens showed reduced susceptibility to clindamycin. None of the isolated bacteria showed resistance to tazobactam and piperacillin.

In their systematic review by Blatt et al. (15) concluded that cefazolin seems to be more effective than penicillin and clindamycin as antibiotic prophylaxis in orthognathic surgery, considering only a perioperative dose of EV antibiotic. In addition, of the 16 studies that evaluated antibiotic prophylaxis in cases of maxillofacial trauma, ampicillin/sulbactam was an adequate antibiotic and seemed to be superior to clindamycin.

A limitation is the bias inherent to an observational study. Moreover, all patients who could have been affected by other factors regarding the appearance of postoperative complications, such as those with decompensated comorbidities, intraoperative incidents, and antibiotic discontinuation, among others, were excluded.

## Conclusions

The clinical results of this retrospective case study are consistent with the literature. Indeed, the bacterial culture results correlate with bacterial resistance to clindamycin and the percentages of patients who developed an SSI when using this antibiotic as prophylaxis in orthognathic surgery. MIs are dynamic in nature and need to be treated by specialists in an adequate health care complex. New scientific evidence is needed to provide specific protocols and guidelines for antibiotic prophylaxis for patients who undergo orthognathic surgery and are allergic to penicillins.

## Figures and Tables

**Table 1:** Table Gender distribution.

Gender	Number of patients
Male	48
Female	52
Total	100

1

**Table 2:** Table Percentage of those allergic to penicillins.

Allergic	% patients
No	98%
Yes	2%

2

**Table 3:** Table Distribution of dentomaxillary anomalies accordingto class

Class	% patients
Skeletal class II	59%
Skeletal class III	41%

3

**Table 4:** Table Percentage of need for surgical revision dependingon type of antibiotic administered.

Antibiotic	Number of patients	Need for surgical review
Cefazolin	98	0%
Clindamycin	2	100%

4

## Data Availability

Declared none.
